# Dose reduction and discontinuation of antipsychotics in psychotic disorders: a systematic review of qualitative studies and meta-synthesis

**DOI:** 10.1038/s41537-026-00747-w

**Published:** 2026-04-08

**Authors:** Sofia Francesca Aprile, Alessandro Rodolico, Antonio Munafò, Irene Bighelli, Filippo Caraci, Stefan Leucht

**Affiliations:** 1 Department of Educational Sciences, Section of Psychology, University of Catania, Catania, Italy; 2 Department of Psychiatry and Psychotherapy, Technical University of Munich, School of Medicine and Health, Department of Psychiatry and Psychotherapy, Klinikum rechts der Isar, Munich, Germany; 3 German Center for Mental Health, Munich-Augsburg Partner site, Munich, Germany; 4 Department of Clinical and Experimental Medicine, Psychiatry Unit, University of Catania, Catania, Italy; 5 Headache Center and Clinical Pharmacology Unit, Careggi University Hospital, Florence, Italy; 6 Department of Neurosciences, Psychology, Drug Research and Child Health, University of Florence, Florence, Italy; 7 Department of Drug and Health Sciences, University of Catania, Catania, Italy; 8 Oasi Research Institute - IRCCS, Unit of Neuropharmacology and Translational Neurosciences, Troina, Italy

**Keywords:** Psychosis, Schizophrenia

## Abstract

Antipsychotic dose reduction and discontinuation are increasingly discussed in the management of psychotic disorders, yet clinical decisions remain challenging and evidence on lived experiences is fragmented. This systematic review and meta-synthesis aimed to synthesize qualitative evidence on subjective experiences of antipsychotic dose reduction and discontinuation among people with psychotic disorders, clinicians, and caregivers. A systematic search was performed on PubMed, Scopus, and PsycINFO. Peer-reviewed qualitative studies exploring subjective experiences with antipsychotic dose reduction and/or discontinuation in subjects with affective and non-affective psychotic disorders were included. Findings were integrated using thematic synthesis. A total of 13 studies were included. Eight studies focused on patients (*N* = 431), three on clinicians (*N* = 86), and two on caregivers (*N* = 26). From subjects’ lived experiences, adverse effects were frequently reported as a driver for considering dose reduction, while concerns about relapse and the effort required to maintain stability contributed to ambivalent or cautious attitudes. Caregivers often reported unfavorable views, expressing fear of symptom recurrence, emotional distress, and risks to relational safety. Clinicians highlighted limited evidence and insufficient resources to support safe, long-term tapering and described uncertainty intensified by negative prior experiences and concerns about professional accountability in the event of relapse. Antipsychotic dose reduction and discontinuation are experienced as complex, preference-sensitive processes shaped by competing priorities and high perceived risks. The findings support the need for triadic shared decision-making that includes caregivers and for the development of structured, case-specific tapering protocols supported by adequate clinical resources and long-term monitoring.

## Background

Antipsychotic drugs are a cornerstone of treatment for schizophrenia spectrum and other psychotic disorders, and long-term maintenance has shown robust evidence for symptom control and relapse prevention^1,2^. Antipsychotics are also widely used in affective psychoses, including bipolar disorder with psychotic features and psychotic depression, both in acute treatment and in maintenance stages alongside mood stabilizers and/or antidepressants^3,4^. However, despite their efficacy in symptom control, antipsychotics are frequently associated with a broad range of adverse effects that might impair daily functioning, social participation, and overall quality of life^5,6^. Common adverse effects of antipsychotics include cardiometabolic disturbances, sedation, extrapyramidal symptoms, hyperprolactinaemia, sexual dysfunctions, and anticholinergic^7^. Notably, antipsychotic-associated weight gain may also worsen psychological well-being through reduced self-esteem, body-image concerns, and stigma^8^. Some evidence suggests that prolonged antipsychotic exposure may be associated with reductions in brain volume, although the extent to which this reflects a medication effect versus illness-related and other confounding factors remains debated, and the overall interpretation of the findings is still controversial^9^. Antipsychotic dose reduction and discontinuation remain a debated clinical strategy, because the potential benefits of lowering medication burden must be weighed against the consistently observed risk of relapse^10,11^. Across discontinuation studies, relapse rates after stopping antipsychotics are high, and dose-reduction approaches generally show the same pattern: the lower the dose, the higher the relapse risk^12,13^. Meta-analytic evidence consistently shows that maintenance antipsychotic treatment is associated with substantially lower relapse risk than discontinuation, making relapse prevention a central consideration in any discussion of tapering^11^. In routine acute-care settings, prescribing is often shaped by immediate safety needs and service constraints, which may encourage dose escalation or antipsychotic polypharmacy, for example, when managing suicidality or aggression, when limited bed capacity and cost pressures shorten admissions, and when early non-response is common^14^. Dose reduction is often pursued to limit dose-dependent adverse effects. Because antipsychotic efficacy is generally achieved within an estimated striatal D2 occupancy “window”, while higher occupancy at higher doses increases extrapyramidal risk, lowering the dose may reduce overall side-effect burden^15,16^. Available trials suggest a trade-off between short- and long-term outcomes: maintenance treatment appears to reduce relapse in the short term, while early dose reduction or discontinuation may carry a higher early relapse risk but has been linked to more favorable functional outcomes at longer follow-up^17,18^. Recent randomized and meta-analytic evidence suggests a possible time-dependent trade-off, with higher relapse risk early after reduction, and mixed or modest differences in functioning later, so dose reduction is best framed as an individualized risk–benefit decision rather than a uniformly beneficial strategy^19^. Even when evidence exists, quantitative studies primarily estimate outcomes such as relapse, symptom change and side effects, but they can miss how stakeholders interpret these outcomes, negotiate risk, and make decisions in everyday life. In this framework, qualitative research might highlight that extend beyond pharmacological outcomes and it has shown its value in capturing patients’ experiences and perceptions of antipsychotic treatment, elements that can substantially shape adherence and treatment success^20^. Qualitative evidence is therefore crucial to understand the lived experience of dose reduction and discontinuation, including perceived changes in well-being, functioning, identity, and relationships, as well as barriers and facilitators within clinical care. This is particularly relevant given that shared decision-making is increasingly recognized as a cornerstone of person-centered care and recovery-oriented treatment management in severe mental illness, yet is often inconsistently implemented in routine psychiatric practice^21,22^. To address this gap, we conducted a systematic review of qualitative studies exploring subjective experiences with antipsychotic dose reduction and discontinuation among people with psychotic disorders, clinicians treating psychosis, and caregivers/family members of people with psychosis.

## Methods

### Search strategy

This systematic review was conducted and reported in accordance with the Enhancing Transparency in Reporting the Synthesis of Qualitative Research (ENTREQ) guidelines^23^. A systematic search was performed in PubMed, Scopus, and PsycINFO. Grey literature was excluded to minimize variability in methodological reporting and to facilitate quality appraisal; therefore, we limited eligible studies to peer-reviewed journal articles, which generally follow recognized qualitative research standards. The search took place on November 1^st^, 2025. The search strategy combined Medical Subject Headings (MeSH) with free-text keywords to maximize the capture of relevant qualitative studies. An example of the database-specific search syntax, adapted to each platform’s indexing, is provided in the supplementary materials (Appendix A). A complete list of excluded studies and the reason for exclusion is reported in Appendix B (Table B.1). The review protocol was registered in PROSPERO (ID: CRD420251235409).

### Eligibility criteria

The following criteria were applied for inclusion in this review:

- Population: we included studies involving (i) adults (≥18 years) with a diagnosis of a non-organic psychotic disorder, including affective and non-affective psychoses (schizophrenia, schizoaffective disorder, schizophreniform disorder, delusional disorder, brief psychotic disorder, drug-induced psychosis, bipolar disorder type I and II, and major depressive disorder with psychotic features), defined according to any recognized diagnostic criteria, and/or (ii) caregivers of such individuals, and/or (iii) mental health professionals (including, but not limited to, psychiatrists, psychologists, mental health nurses). We excluded studies focused on children or adolescents (<18 years) and those addressing organic psychosis.
- Phenomenon of interest: this review did not evaluate the effects of a specific intervention. Instead, we included qualitative studies that explored experiences, perspectives, beliefs, decision-making processes, and contextual factors related to antipsychotic dose reduction and/or discontinuation among eligible service users, caregivers, and clinicians.
- Comparators: no comparator or control group was required for inclusion.
- Design: we included any primary study using qualitative methods for data collection and analysis, such as semi-structured or in-depth interviews, focus groups, or open-ended survey responses. Both randomized and nonrandomized study types were eligible if they reported relevant qualitative findings. We excluded literature reviews.
- Context: we did not restrict eligibility by recruitment setting or geographical location.

### Data extraction

Study screening and selection were conducted independently by SFA and AM, with disagreements resolved through discussion and, if needed, adjudication by a third researcher (AR). Following ENTREQ guidance, we appraised included studies using the CASP Qualitative Checklist^24^, a 10-item tool for identifying methodological strengths and limitations. Quality appraisal was completed independently by SFA and AR, with consensus used to resolve disagreements. Overall CASP ratings are reported in Table 1, and item-level (yes/no) judgements are provided in the supplementary materials. When required, we contacted corresponding authors for additional details on antipsychotic treatment in study samples.

**Table 1:** Characteristics of included studies^a^.

Study	Country	Aim	Design	Setting	Population	Diagnosis	Sample size (patients)	Sample size (clinicians)	Sample size (caregivers)	Qualitative data analysis	Age mean (SD)	CASP
Béchar et al.^34^	Canada	To explore the decision-making process leading to discontinuation in patients with FEP	Qualitative (semi-structured interviews)	Early intervention services	Patients (FEP)	SSDs, BD, MDD with psychosis	12	0	0	Thematic analysis	26.25 (5.1)	10
Cooper et al.^35^	UK	To explore mental health practitioners’ experiences and attitudes towards antipsychotic reduction and discontinuation	Qualitative (focus groups)	Mental health clinics	Clinicians	NA	0	35	0	Thematic analysis	43 (8.3)	10
Crellin et al.^28^	UK	To explore patients’ views about antipsychotics dose reduction or discontinuation	Mixed-methods (face-to-face interviews with fixed and open-ended question; DAI-10)	Community mental health services	Patients	SSDs	269 (non-affective psychosis)	0	0	Content analysis	46.2 (11.50)	8
Forsberg et al.^36^	UK	To explore clinicians’ perspectives on supporting antipsychotics discontinuation	Qualitative (in-depth interviews)	NHT trusts	Clinicians	NA	0	12	0	Grounded theory	42.8 (6.9)	9
Gates et al.^29^	Australia	To explore the experience of participating in an antipsychotic dose reduction trial	Qualitative (semi-structured interviews)	Orygen’s Early Psychosis Prevention and Intervention Center	Patients (FEP)	SSDs, BD type I, MDD with psychosis	5 (*N* = 3 non-affective psychosis; *N* = 2 affective psychosis)	0	0	Interpretative Phenomenological Analysis (IPA)	23 (1)	10
Geyt et al.^32^	UK	To explore personal accounts of discontinuing antipsychotics	Qualitative (semi-structured interviews)	Participants’ home	Patients	Non-organic psychosis	12	0	0	Grounded theory	NR	9
Larsen-Barr & Seymour, 2021^53^	New Zealand	To explore wellbeing after successful antipsychotics withdrawal	Qualitative (semi-structured interviews)	Phone calls or face-to-face interviews (participants living in the community)	Patients	BD, SSDs, MDD, OCD	7 (*N* = 3 affective psychosis)	0	0	Thematic analysis	50.29 (8.32)	9
Lewins et al.^38^	UK	To explore long-term use, reduction and discontinuation of antipsychotics from caregivers’ perspectives of people with SSDs	Qualitative (semi-structured interviews)	Community mental health services	Caregivers	SSDs	0	0	11	Thematic analysis	NR	10
Morant et al., ^30^	UK	To explore antipsychotics dose reduction and discontinuation in the context of a RCT	Qualitative (semi-structured interviews)	Community mental health services	Patients	SSDs	26 (non-affective psychosis)	0	0	Thematic analysis	NR	9
Nøstdal et al.^33^	Denmark	To explore schizophrenia patients’ experiences with dose reduction	Qualitative (open-ended survey)	Outpatient clinic	Patients	Schizophrenia	88 (non-affective psychosis)	0	0	Quantitative content analysis	39.2 (11.6)	8
Orlando et al.^39^	UK	To explore caregivers and family experiences of dose reduction and discontinuation in the context of a reduction RCT	Qualitative (semi-structured interviews)	Research centers	Caregivers	SSDs	0	0	15	Thematic analysis	NR	10
Roed et al.^37^	Denmark	To explore mental health staff’s perspectives on antipsychotics tapering	Qualitative (focus groups)	Mental health services	Clinicians	NA	0	39	0	Thematic analysis	45	9
Southern et al.^31^	UK	To explore patients’ experience with clozapine discontinuation	Qualitative (semi-structured interviews)	Participants’ home or clinical team base	Patients	SSDs	16 (non-affective psychosis)	0	0	Grounded theory	46.94 (12.78)	10

^a^Reported themes from each included study are summarized in the supplementary materials (Table C.3). FEP: first episode psychosis.

### Data analysis

Data coding and analysis were conducted by SFA and reviewed by AM, with interpretive differences discussed to agreement. We used thematic synthesis to integrate qualitative evidence on healthcare experiences and views^25^, drawing on meta-ethnography and grounded theory concepts^26,27^. This approach supports cross-study integration, hypothesis generation for later quantitative work, and conceptual saturation. The synthesis followed three steps: (1) line-by-line coding of relevant data from each study, (2) grouping related codes into descriptive themes, and (3) interpreting these into analytical themes that provide higher-level explanations and implications. No qualitative software was used.

## Results

**Fig. 1:**
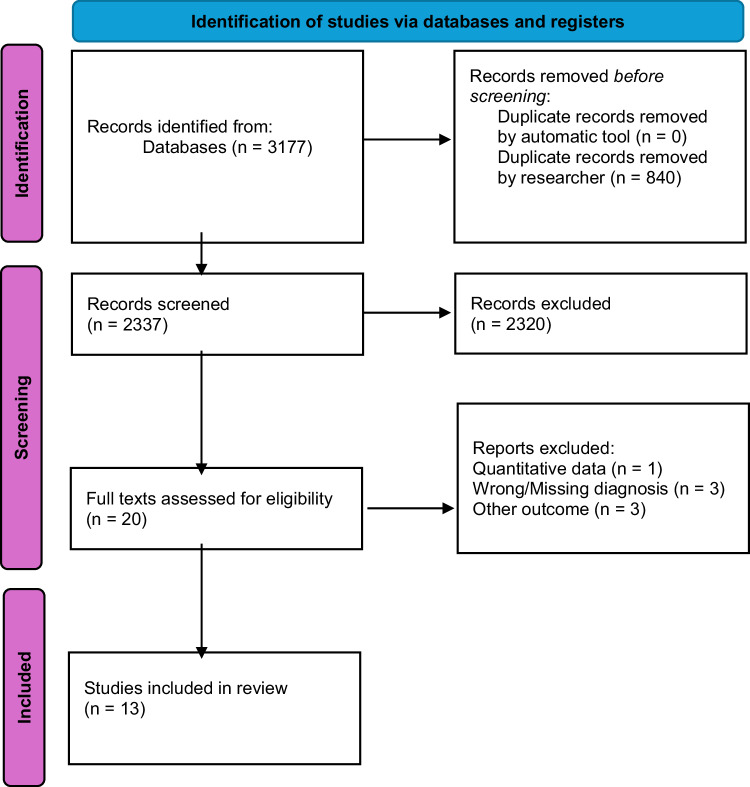
Preferred Reporting Items for Systematic reviews and Meta-Analyses (PRISMA) flow chart illustrating the systematic selection process.

Twenty studies were retrieved for full-text screening, of which 13 were included, while 7 were excluded (Fig. 1). Among the included studies, 8 focused on patients (*N* = 431), 3 on clinicians (*N* = 86), and 2 on caregivers of people with affective or non-affective psychosis (*N* = 26). Other relevant characteristics of the sample are reported in Table 1. Overall, the main qualitative themes appeared broadly consistent across studies including SSDs only and those including mixed samples with affective psychoses.

### Qualitative synthesis of reported studies

Line-by-line codes were grouped into descriptive themes, which were then interpreted into higher-order analytical themes. Findings from subsequent studies were mapped onto existing concepts, with new ones created when necessary. Key synthesis results are summarized in Table 2 and detailed below.

**Table 2:** Thematic analysis of subjective experiences of antipsychotic dose reduction and discontinuation from the perspectives of patients, clinicians, and caregivers.

Analytical theme	Descriptive theme	Example text
*Patients*		
Barriers of tapering	Fear of relapse	“I functioned as an average human being, as a normal human being. But I also had this time bomb ticking away underneath me where I feared a psychosis rebound”^30^
	Benefits of long-term treatment	“It actually helped me in more ways than anything else had… used to make me happy…felt annoyed with myself because I can’t get back to what I was on Clozaril®…it actually saved me from all the voices and that, and I’m a wreck now”^31^
	Emotional difficulties and negative consequences	“The voices got very bad, I got very paranoid”^31^
Facilitators of tapering	Burden of medication: side effects, impairment, long-term effects	“I’ve had stuff that’s given me a condition called Wolff-Parkinson-White syndrome, where my heart’s had an irregular beat”^32^.
	Identity goals: independence, living a normal life, testing recovery	“I want to be more awake and present. I want to be free of medicine. I want to get an education. I want to have a normal life”^33^.
	Support, trust and collaborative care	“Having the support of my doctor who didn’t take a hard line with me and say “you must go back on your medication,” that in itself was very helpful”^32^.
	Positive outcomes of reduction	“I persevered and it worked… yes, a bit shaky at first [after reducing] but that’s all gone away”^30^
Unsafe tapering	Limited knowledge, lack of trust, alternative interpretations	“They gave me a little leaflet… but it didn’t tell me all the nasty side effects I’ve experienced since I’ve been on it”^32^.
	Stigma driving covert discontinuation	“I want to be normal people, like you know, I don’t want to take—normal people don’t take medicine or anything”^29^
	Self-directed stopping and non-adherence patterns	“I tried like two, three days I did not take medicine, but it showed me some problems”^29^.
	Tapering without adequate support	“If I told my mum, who lives nearly 200 miles away, she’d have panicked”^32^
*Clinicians*		
Practical feasibility	Service constraints	“That is the thing that I need to know is that…it’s not that the client [who is reducing] would be left hanging there without having any resource, any back-up. Because you try to get an appointment with the consultant—it’s difficult enough as it is”^35^.
	Limited evidence	“We don’t actually know whether it is better to reduce or not, and therefore, whilst there might be a general idea that reducing is a good idea, without the evidence it is not unethical to continue at what you anticipate as being a stable dose, because we do not actually know that that’s worse”^35^.
Harm reduction and dose optimization	Minimal effective dose and tolerability	“So sometimes it’s worth considering: is it better to have a smaller dose and that person will better adhere to the medication, rather than having too high a dose and the side-effects will just make them stop, you know?”^30^
	Collaborative tapering	“You know, if you actually—people feel that you know—you’ve said to them, you said well look, we probably can’t stop this, but let’s have a look—let’s try a small reduction and see what happens, if that goes okay then we can, you know, and if you can feel you’re working with them, it improves the relationship as well”^35^.
	Recovery	“Some recover by getting off the medication and some can recover and become what they want with some amount of medication. […] None of us can say when that is. But if you feel good about yourself and the world around you, then you are recovered”^37^.
Relapse prevention and risk management	Fear of being blamed	“You can almost hear someone saying to you, ‘you allowed this to happen, you were part of it, why did you do that? Why did you let them have that choice?’ That kind of thing. So I can always imagine like a relative saying it, or you know, someone in an SUI [Serious Untoward Incident], an investigation saying ‘Why would you do that?”^36^
	Risks of discontinuation	“From my own personal experience, I’ve got to say everyone that I’ve tried to stop, all relapsed and all become unwell”^35^.
	Prevention and maintenance	“The medication, I see it also as a sort of preventative thing for the future and it has been shown that essentially if you keep people on these medications it not only prevents them relapsing but it also protects their brains as it were, and protects them for their…improves their future functioning”^35^.
*Caregivers*		
Safeguarding stability and preventing relapse	Fear of relapse	“My opinion is: once you’ve stabilized something, you can’t play about with it- that’s it. From my experience with changing the medication, it can go very wrong”.^38^
	Managing adherence	“I have trouble getting her to take them sometimes… So I keep reminding her that the doctor said she’s got to do it”.^38^
	Cautious change and close monitoring	“I don’t know what effect it could have if she tries to reduce the tablets more than what she has done already… I’m cautiously optimistic – it can help, but I don’t know”.^39^
Antipsychotics benefits against burdens	Recovering and functioning	“When he takes the meds, he’s back… he comes back. “Mum give me a hug”, all those things”.^38^
	Side effects burden	“He was just like a zombie– sitting in the chair dribbling and dribbling. It wasn’t a quality of life. And I thought, oh I wonder if he’ll always be like this”.^38^
Caregivers’ responsibilities and burdens	Personal impact	“I get a bit frightened… I don’t want him to start acting up, misbehaving and things like that, it’s too much for me”.^39^
	Observed outcomes	“Since she’s been off of them [antipsychotics] she’s got a lot more bubbly… Her way of looking at things is changing, the way she thinks is changing, the way she speaks to people is changing”.^39^

### Patients’ perspectives on antipsychotics dose reduction and discontinuation

Patients described a range of experiences that underscored both perceived benefits and drawbacks of tapering. Those opposed to dose reduction emphasized the value of long-term antipsychotic treatment in enabling a return to premorbid functioning and maintaining everyday activities that they felt would not be possible without medication^28,29^. Others attributed their reluctance to prior attempts at reduction that were followed by symptom re-emergence^28,30,31^. Even in the absence of a clear relapse, fear of recurrence was often sufficient to deter further attempts to taper^30^. Regarding potential facilitators, participants described motivations such as regaining independence, receiving support from others, experiencing burdensome antipsychotic side effects, and noticing perceived benefits after dose reduction^28–33^. Participants identified antipsychotic side effects, such as weight gain, sedation, low energy, emotional blunting, sexual dysfunction, constipation, and extrapyramidal symptoms, as key motivators for attempting dose reduction or discontinuation^28,29,31–33^. A further challenge related to unsafe tapering practices. Many participants described attempts to stop medication on their own, which were often followed by relapse^33^. These decisions were frequently linked to the patient–clinician relationship: some participants felt they had not been given sufficient information about adverse effects, while others pursued discontinuation as a way to regain a sense of control and autonomy^32^. Notably, the sub-theme of stigma driving covert discontinuation emerged specifically in the studies including first-episode psychosis populations, suggesting that concerns about identity, disclosure, and being labeled may be particularly salient at earlier stages of illness^32,34^.

### Clinicians’ perspectives on antipsychotics dose reduction and discontinuation

Several clinicians, although not explicitly opposed to tapering, expressed concerns about practical barriers. They pointed to limited local resources to provide long-term follow-up and monitoring; some viewed dose reduction as risky without assurance that patients could be adequately supervised and therefore preferred to maintain stable doses^35^. Patients’ poor adherence was also identified as a challenge, and they pointed out that the lack of clear guidance in the literature may further complicate decision-making^35^. Other participants opposed dose reduction, stating that, based on their clinical experience, tapering attempts ultimately led to relapse, and therefore viewed the practice as best avoided and unnecessarily risky both for patients and clinicians themselves^35,36^. Some clinicians actively sought to discourage dose reduction, emphasizing that long-term pharmacotherapy has a prophylactic effect and lowers the risk of relapse^35^. Other clinicians acknowledged that adopting a rigid stance, particularly when patients are experiencing adverse effects, may be counterproductive and could prompt abrupt self-discontinuation. They emphasized the importance of listening to patients’ concerns and collaborating to identify an individualized optimal dose^35,37^.

### Caregivers’ perspectives on antipsychotics dose reduction and discontinuation

From caregivers’ perspectives, many were not supportive of dose reduction, preferring to protect the hard-won stability achieved with treatment rather than risk relapse through tapering^38^. Regarding long-term antipsychotic treatment, caregivers expressed mixed views: some emphasized the benefits achieved and were reluctant to make changes, noting that over time their loved ones had “returned to their former selves,” whereas others highlighted the distress associated with medication side effects^38,39^. Others were hesitant to alter the dosage because of the intense stress and emotional toll of caring for a relative with psychosis and the demands that this caregiving role entails^38,39^.

### Assessment of quality and methodological rigor

Methodological quality was assessed using the CASP tool. Included studies scored between 8 and 10, indicating good overall methodological rigor across the evidence base. The most frequently unmet criterion was CASP item 6, which assesses whether studies considered and reported the potential influence of the interviewer–participant relationship. Half of the included studies (53.8%) did not report this information.

## Discussion

This is the first systematic synthesis specifically focused on qualitative evidence regarding antipsychotic dose reduction in psychotic disorders. A previous review, which included different mental health diagnoses and did not apply restriction on the type of psychotropic medications, suggested that self-discontinuation, often attributed to poor insight, is frequently better understood as an individual cost–benefit appraisal of continuing versus stopping treatment^40^. Consistent with prior qualitative findings, our synthesis indicates that multiple factors might motivate antipsychotic dose reduction. The most frequently discussed driver in the literature is the burden of adverse effects, which is well known to influence adherence in psychiatric populations^41^. Beyond adverse effects, the wish to reduce antipsychotic treatment may also reflect a desire to regain autonomy and to distance oneself from a patient identity associated with long-term psychiatric care. In this sense, dose reduction may carry a symbolic meaning that extends beyond medication burden alone, being linked to agency, self-definition, and the hope of moving beyond illness. Tapering may therefore represent not only a treatment decision, but also an attempt to renegotiate identity beyond the role of psychiatric patient. Although such expectations may not always correspond to clinical realities, they remain highly relevant to the decision-making process and underscore the importance of exploring with patients what dose reduction represents for them personally. Views were mixed, and dose reduction was not universally endorsed. Some participants expressed strong reservations, highlighting concerns about relapse and underscoring the effort required to reach a stable and balanced state^29^. Taken together, these accounts suggest an asymmetry in risk perception, whereby the possibility of relapse may be weighted more heavily than the burden of adverse effects. This may help explain why dose reduction is often approached cautiously even when treatment-related side effects are substantial. These reflections from individuals with lived experience of illness raise an important question: although clinicians are guided by the principle of *primum non nocere*, this does not necessarily imply that dose reduction is always the least harmful option. When side effects are disabling or treatment is experienced as burdensome, dose reduction may be an appropriate therapeutic option. However, it is essential to explore in depth whether this truly aligns with the patient’s wishes. Conversely, if a person wishes to continue treatment because they do not experience adverse effects, or because they prioritize functioning and stability over potential side effects, the same principle of non-maleficence should discourage initiating an unwanted tapering process, given the possible increased risk of relapse^42^. The caregiver sample also reported, in most cases, unfavorable opinions regarding dose reduction, often expressing concerns about symptom recurrence and the fear of “losing” their loved ones again, as well as being hurt^38,39^. These findings highlight the importance of shared decision-making, including caregivers, particularly as some reported not being sufficiently involved before tapering was initiated^39^. Triadic shared decision-making has been described as beneficial for both patients and clinicians^43,44^. Caregiver involvement can provide emotional support to patients while also offering clinicians valuable information. However, this approach remains insufficiently implemented in routine psychiatric practice^45,46^. Improving communication with people experiencing psychosis and their families is essential to address the information gaps and unmet informational needs they frequently report^20^. Regarding clinicians’ experiences, views were similarly mixed and often conflicting. Clinicians mainly highlighted two challenges: limited evidence to guide practice and insufficient resources to support a safe, long-term dose reduction process^35,37^. Taken together with negative clinical experiences, these factors contribute to considerable uncertainty among clinicians. Even when tapering may be appropriate, concerns about professional consequences in the event of relapse may discourage clinicians from attempting it^36^. This hesitation may reflect not only clinical uncertainty, but also the psychological burden of responsibility in situations where relapse may be experienced as preventable and professionally consequential. This highlights an important take-home message: expanding the evidence base on dose reduction is necessary, but not sufficient, in psychiatric research. Several structured approaches to antipsychotic dose reduction have already been proposed, including gradual tapering with pre-specified schedules^47,48^, clinician-guided discontinuation based on symptom monitoring and patient preferences^18,49^, hyperbolic tapering grounded in receptor-occupancy pharmacodynamics^50,51^, and biomarker-guided dose titration using plasma-level or D₂-occupancy modeling^52^. Despite these proposals, there remains no clear consensus regarding the optimal schedule, pace of reduction, or stopping criteria in routine clinical practice. Future research should focus on validating and comparing these existing approaches head-to-head, and on developing safeguards that support clinicians and caregivers throughout the process.

### Limitations and strengths

This study has some limitations. The analysis was based only on quotations reported in published papers, without access to full transcripts, which may have reduced contextual accuracy. As with all qualitative synthesis, interpretations may also be influenced by researcher subjectivity despite efforts to ensure rigor. The multidisciplinary backgrounds of the review team, including clinical psychology, psychiatry, and pharmacology, may have shaped the interpretation of findings; to enhance reflexive transparency, interpretive disagreements were discussed within the team until consensus was reached. The included studies involved diagnostically heterogeneous populations, including SSDs and affective disorders; as the role of antipsychotics and the clinical implications of dose reduction may differ across these groups, findings should be interpreted with caution. The studies were also heterogeneous in terms of illness stage and treatment context, including FEP samples and clozapine-related discontinuation contexts, which should also be considered when interpreting the findings. Finally, subgroup analyses by specific treatment were not feasible due to missing data, highlighting a gap in the literature. At the same time, the study has important strengths. It integrates patients’, caregivers’, and clinicians’ perspectives on antipsychotic dose reduction, highlighting key barriers and unmet needs in implementing tapering in routine practice. Moreover, the included studies achieved high CASP scores, supporting the quality and robustness of the findings.

## Conclusion

Our systematic review focused on qualitative findings about antipsychotics dose reduction and discontinuation in subjects with psychotic disorders, clinicians and caregivers. Our study highlights that tapering is experienced as a complex, highly individual process shaped by a careful weighing of benefits and risks. While adverse effects frequently motivate attempts to taper, substantial concerns about relapse, loss of stability, and the emotional burden on families contribute to mixed and often cautious attitudes among patients, caregivers, and clinicians. However, tapering should not be considered appropriate for all patients, and maintenance treatment remains a strongly evidence-supported strategy for relapse prevention in many individuals with psychotic disorders. When dose reduction is considered, our findings underscore the need for dose reduction decisions to be embedded within a triadic shared decision-making model that actively involves caregivers, with tapering protocols and clinical resources structured to support this process. Future research should move beyond establishing efficacy alone and prioritize the development and testing of structured, case-specific tapering pathways with long-term monitoring to ensure safety, feasibility, and acceptability in routine care.

## Supplementary information

Supplementary materials

## Data Availability

The data that support the findings of this study are available from the corresponding author upon reasonable request.
