# Supplementary material for: Dose reduction and discontinuation of antipsychotics in psychotic disorders: a systematic review of qualitative studies and meta-synthesis
Source: Schizophrenia (Heidelb). 2026 Apr 8;12(1):37. doi: 10.1038/s41537-026-00747-w (PMC13068915; doi:10.1038/s41537-026-00747-w)
Supplement: Supplementary file 1 — Supplementary materials [file 41537_2026_747_MOESM1_ESM.docx]

**Supplementary Materials**

**Appendix A**

**Search strategy**: #1 AND #2 AND #3

#1: "chronic"[Title/Abstract] OR "chronically"[Title/Abstract] OR "continuance"[Title/Abstract] OR "continue"[Title/Abstract] OR "continued"[Title/Abstract] OR "continuing"[Title/Abstract] OR "continuous"[Title/Abstract] OR "extended"[Title/Abstract] OR "longterm"[Title/Abstract] OR "long-term"[Title/Abstract] OR "maintenance"[Title/Abstract] OR "maintain"[Title/Abstract] OR "maintained"[Title/Abstract] OR "maintaining"[Title/Abstract] OR "maintains"[Title/Abstract] OR "ongoing"[Title/Abstract] OR "persisting"[Title/Abstract] OR "persistent"[Title/Abstract] OR "prophylactic"[Title/Abstract] OR "prophylaxis"[Title/Abstract] OR "sustained"[Title/Abstract] OR "relapse prevention"[Title/Abstract] OR "adapt"[Title/Abstract] OR "adapted"[Title/Abstract] OR "adapting"[Title/Abstract] OR "adjust"[Title/Abstract] OR "adjusted"[Title/Abstract] OR "adjusting"[Title/Abstract] OR "adjustment"[Title/Abstract] OR "cease"[Title/Abstract] OR "ceased"[Title/Abstract] OR "ceasing"[Title/Abstract] OR "cessation"[Title/Abstract] OR "cut"[Title/Abstract] OR "cutting"[Title/Abstract] OR "de-escalate"[Title/Abstract] OR "de-escalated"[Title/Abstract] OR "de-escalating"[Title/Abstract] OR "de-escalation"[Title/Abstract] OR "decrease"[Title/Abstract] OR "decreased"[Title/Abstract] OR "decreasing"[Title/Abstract] OR "deprescribe"[Title/Abstract] OR "deprescribed"[Title/Abstract] OR "deprescribing"[Title/Abstract] OR "deprescription"[Title/Abstract] OR "diminish"[Title/Abstract] OR "diminished"[Title/Abstract] OR "diminishing"[Title/Abstract] OR "discontinuance"[Title/Abstract] OR "discontinuation"[Title/Abstract] OR "discontinue"[Title/Abstract] OR "discontinued"[Title/Abstract] OR "discontinuing"[Title/Abstract] OR "dismiss"[Title/Abstract] OR "dismissed"[Title/Abstract] OR "dismissing"[Title/Abstract] OR "dismission"[Title/Abstract] OR "down titrate"[Title/Abstract] OR "down titrated"[Title/Abstract] OR "down titrating"[Title/Abstract] OR "down titration"[Title/Abstract] OR "downsize"[Title/Abstract] OR "downsized"[Title/Abstract] OR "downsizing"[Title/Abstract] OR "drop"[Title/Abstract] OR "dropped"[Title/Abstract] OR "dropping"[Title/Abstract] OR "end"[Title/Abstract] OR "ending"[Title/Abstract] OR "halt"[Title/Abstract] OR "halted"[Title/Abstract] OR "halting"[Title/Abstract] OR "interrupting"[Title/Abstract] OR "interruption"[Title/Abstract] OR "lessen"[Title/Abstract] OR "lessened"[Title/Abstract] OR "lessening"[Title/Abstract] OR "lower"[Title/Abstract] OR "lowered"[Title/Abstract] OR "lowering"[Title/Abstract] OR "minified"[Title/Abstract] OR "minify"[Title/Abstract] OR "minifying"[Title/Abstract] OR "minimization"[Title/Abstract] OR "minimize"[Title/Abstract] OR "minimized"[Title/Abstract] OR "minimizing"[Title/Abstract] OR "optimization"[Title/Abstract] OR "optimize"[Title/Abstract] OR "optimized"[Title/Abstract] OR "optimizing"[Title/Abstract] OR "pause"[Title/Abstract] OR "paused"[Title/Abstract] OR "pausing"[Title/Abstract] OR "quit"[Title/Abstract] OR "quitted"[Title/Abstract] OR "quitting"[Title/Abstract] OR "reduce"[Title/Abstract] OR "reduced"[Title/Abstract] OR "reducing"[Title/Abstract] OR "reduction"[Title/Abstract] OR "shrink"[Title/Abstract] OR "shrinking"[Title/Abstract] OR "shrunk"[Title/Abstract] OR "shrunken"[Title/Abstract] OR "simplification"[Title/Abstract] OR "simplified"[Title/Abstract] OR "simplify"[Title/Abstract] OR "simplifying"[Title/Abstract] OR "stop"[Title/Abstract] OR "stoppage"[Title/Abstract] OR "stopped"[Title/Abstract] OR "stopping"[Title/Abstract] OR "streamline"[Title/Abstract] OR "streamlined"[Title/Abstract] OR "streamlining"[Title/Abstract] OR "suspend"[Title/Abstract] OR "suspending"[Title/Abstract] OR "suspension"[Title/Abstract] OR "tailor"[Title/Abstract] OR "tailored"[Title/Abstract] OR "tailoring"[Title/Abstract] OR "taper"[Title/Abstract] OR "tapered"[Title/Abstract] OR "tapering"[Title/Abstract] OR "terminate"[Title/Abstract] OR "terminated"[Title/Abstract] OR "terminating"[Title/Abstract] OR "termination"[Title/Abstract] OR "withdraw"[Title/Abstract] OR "withdrawal"[Title/Abstract] OR "withdrawing"[Title/Abstract] OR "withdrew"[Title/Abstract]

#2: Pimavanserin[tiab] OR Brexpiprazole[tiab] OR Cariprazine[tiab] OR Iloperidone[tiab] OR Paliperidone[tiab] OR Aripiprazole[tiab] OR Zotepine[tiab] OR Mosapramine[tiab] OR Risperidone[tiab] OR lithium[tiab] OR Levosulpiride[tiab] OR Veralipride[tiab] OR Amisulpride[tiab] OR Remoxipride[tiab] OR Tiapride[tiab] OR Sultopride[tiab] OR Sulpiride[tiab] OR Samidorphan[tiab] OR Olanzapine[tiab] OR Clothiapine[tiab] OR Asenapine[tiab] OR Quetiapine[tiab] OR Olanzapine[tiab] OR Clozapine[tiab] OR Loxapine[tiab] OR Penfluridol[tiab] OR Pimozide[tiab] OR Fluspirilene[tiab] OR Zuclopenthixol[tiab] OR Thiothixene[tiab] OR Chlorprothixene[tiab] OR Clopenthixol[tiab] OR Flupentixol[tiab] OR Lurasidone[tiab] OR Ziprasidone[tiab] OR Sertindole[tiab] OR Molindone[tiab] OR Oxypertine[tiab] OR Lumateperone[tiab] OR Fluanisone[tiab] OR Droperidol[tiab] OR Benperidol[tiab] OR Bromperidol[tiab] OR Pipamperone[tiab] OR Moperone[tiab] OR Melperone[tiab] OR Trifluperidol[tiab] OR Haloperidol[tiab] OR Pipotiazine[tiab] OR Mesoridazine[tiab] OR Thioridazine[tiab] OR Periciazine[tiab] OR Perazine[tiab] OR Butaperazine[tiab] OR Thioproperazine[tiab] OR Acetophenazine[tiab] OR Trifluoperazine[tiab] OR Thiopropazate[tiab] OR Prochlorperazine[tiab] OR Perphenazine[tiab] OR Fluphenazine[tiab] OR Dixyrazine[tiab] OR Chlorproethazine[tiab] OR Cyamemazine[tiab] OR Triflupromazine[tiab] OR Acepromazine[tiab] OR Promazine[tiab] OR Methotrimeprazine[tiab] OR Chlorpromazine[tiab] OR antipsychotic*[tiab] OR neuroleptic*[tiab] OR "anti dopaminergic*"[tiab] OR antidopaminergic*[tiab] Pimavanserin[tiab] OR Brexpiprazole[tiab] OR Cariprazine[tiab] OR Iloperidone[tiab] OR Paliperidone[tiab] OR Aripiprazole[tiab] OR Zotepine[tiab] OR Mosapramine[tiab] OR Risperidone[tiab] OR lithium[tiab] OR Levosulpiride[tiab] OR Veralipride[tiab] OR Amisulpride[tiab] OR Remoxipride[tiab] OR Tiapride[tiab] OR Sultopride[tiab] OR Sulpiride[tiab] OR Samidorphan[tiab] OR Olanzapine[tiab] OR Clothiapine[tiab] OR Asenapine[tiab] OR Quetiapine[tiab] OR Olanzapine[tiab] OR Clozapine[tiab] OR Loxapine[tiab] OR Penfluridol[tiab] OR Pimozide[tiab] OR Fluspirilene[tiab] OR Zuclopenthixol[tiab] OR Thiothixene[tiab] OR Chlorprothixene[tiab] OR Clopenthixol[tiab] OR Flupentixol[tiab] OR Lurasidone[tiab] OR Ziprasidone[tiab] OR Sertindole[tiab] OR Molindone[tiab] OR Oxypertine[tiab] OR Lumateperone[tiab] OR Fluanisone[tiab] OR Droperidol[tiab] OR Benperidol[tiab] OR Bromperidol[tiab] OR Pipamperone[tiab] OR Moperone[tiab] OR Melperone[tiab] OR Trifluperidol[tiab] OR Haloperidol[tiab] OR Pipotiazine[tiab] OR Mesoridazine[tiab] OR Thioridazine[tiab] OR Periciazine[tiab] OR Perazine[tiab] OR Butaperazine[tiab] OR Thioproperazine[tiab] OR Acetophenazine[tiab] OR Trifluoperazine[tiab] OR Thiopropazate[tiab] OR Prochlorperazine[tiab] OR Perphenazine[tiab] OR Fluphenazine[tiab] OR Dixyrazine[tiab] OR Chlorproethazine[tiab] OR Cyamemazine[tiab] OR Triflupromazine[tiab] OR Acepromazine[tiab] OR Promazine[tiab] OR Methotrimeprazine[tiab] OR Chlorpromazine[tiab] OR antipsychotic*[tiab] OR neuroleptic*[tiab] OR "anti dopaminergic*"[tiab] OR antidopaminergic*[tiab]

#3: "Document Analysis"[MeSH Terms:noexp] OR "focus groups"[MeSH Terms:noexp] OR "interviews as topic"[MeSH Terms:noexp] OR "narration"[MeSH Terms:noexp] OR "qualitative research"[MeSH Terms:noexp] OR "ethnograph*"[Title/Abstract] OR "field work"[Title/Abstract:~1] OR "fieldwork"[Title/Abstract] OR "focus group"[Title/Abstract] OR "focus groups"[Title/Abstract] OR "key informant"[Title/Abstract] OR "key informants"[Title/Abstract] OR "qualitative research"[Title/Abstract] OR "qualitative studies"[Title/Abstract] OR "qualitative study"[Title/Abstract] OR "qualitative synthesis"[Title/Abstract] OR "depth discussion"[Title/Abstract:~2] OR "depth discussions"[Title/Abstract:~2] OR "depth interview"[Title/Abstract:~2] OR "depth interviews"[Title/Abstract:~2] OR "depth questionnaire"[Title/Abstract:~2] OR "depth questionnaires"[Title/Abstract:~2] OR "face discussion"[Title/Abstract:~2] OR "face discussions"[Title/Abstract:~2] OR "face interview"[Title/Abstract:~2] OR "face interviews"[Title/Abstract:~2] OR "face questionnaire"[Title/Abstract:~2] OR "face questionnaires"[Title/Abstract:~2] OR "group discussion"[Title/Abstract:~2] OR "group discussions"[Title/Abstract:~2] OR "group interview"[Title/Abstract:~2] OR "group interviewed"[Title/Abstract:~2] OR "group interviews"[Title/Abstract:~2] OR "group questionnaire"[Title/Abstract:~2] OR "group questionnaires"[Title/Abstract:~2] OR "groups interviewed"[Title/Abstract:~2] OR "guided discussion"[Title/Abstract:~2] OR "guided discussions"[Title/Abstract:~2] OR "guided interview"[Title/Abstract:~2] OR "guided interviews"[Title/Abstract:~2] OR "guided questionnaire"[Title/Abstract:~2] OR "guided questionnaires"[Title/Abstract:~2] OR "indepth discussion"[Title/Abstract:~2] OR "indepth discussions"[Title/Abstract:~2] OR "indepth interview"[Title/Abstract:~2] OR "indepth interviews"[Title/Abstract:~2] OR "indepth questionnaire"[Title/Abstract:~2] OR "indepth questionnaires"[Title/Abstract:~2] OR "semistructured discussion"[Title/Abstract:~2] OR "semistructured discussions"[Title/Abstract:~2] OR "semistructured interview"[Title/Abstract:~2] OR "semistructured interviews"[Title/Abstract:~2] OR "semistructured questionnaire"[Title/Abstract:~2] OR "semistructured questionnaires"[Title/Abstract:~2] OR "structured discussion"[Title/Abstract:~2] OR "structured discussions"[Title/Abstract:~2] OR "structured interview"[Title/Abstract:~2] OR "structured interviews"[Title/Abstract:~2] OR "structured questionnaire"[Title/Abstract:~2] OR "structured questionnaires"[Title/Abstract:~2] OR "unstructured discussion"[Title/Abstract:~2] OR "unstructured discussions"[Title/Abstract:~2] OR "unstructured interview"[Title/Abstract:~2] OR "unstructured interviews"[Title/Abstract:~2] OR "unstructured questionnaire"[Title/Abstract:~2] OR "unstructured questionnaires"[Title/Abstract:~2] OR "Patient Experience"[Title/Abstract:~4] OR "Patient Experiences"[Title/Abstract:~4] OR "Patient Belief"[Title/Abstract:~4] OR "Patient Beliefs"[Title/Abstract:~4] OR "Patient Attitude"[Title/Abstract:~4] OR "Patient Attitudes"[Title/Abstract:~4] OR "Patient Perspective"[Title/Abstract:~4] OR "Patient Perspectives"[Title/Abstract:~4] OR "Patients Experience"[Title/Abstract:~4] OR "Patients Experiences"[Title/Abstract:~4] OR "Patients Belief"[Title/Abstract:~4] OR "Patients Beliefs"[Title/Abstract:~4] OR "Patients Attitude"[Title/Abstract:~4] OR "Patients Attitudes"[Title/Abstract:~4] OR "Patients Perspective"[Title/Abstract:~4] OR "Patients Perspectives"[Title/Abstract:~4] OR "Service User Experience"[Title/Abstract:~4] OR "Service User Experiences"[Title/Abstract:~4] OR "Service User Belief"[Title/Abstract:~4] OR "Service User Beliefs"[Title/Abstract:~4] OR "Service User Attitude"[Title/Abstract:~4] OR "Service User Attitudes"[Title/Abstract:~4] OR "Service User Perspective"[Title/Abstract:~4] OR "Service User Perspectives"[Title/Abstract:~4] OR "Service Users Experience"[Title/Abstract:~4] OR "Service Users Experiences"[Title/Abstract:~4] OR "Service Users Belief"[Title/Abstract:~4] OR "Service Users Beliefs"[Title/Abstract:~4] OR "Service Users Attitude"[Title/Abstract:~4] OR "Service Users Attitudes"[Title/Abstract:~4] OR "Service Users Perspective"[Title/Abstract:~4] OR "Service Users Perspectives"[Title/Abstract:~4] OR "Patient Preference"[Title/Abstract:~4] OR "Patient Preferences"[Title/Abstract:~4] OR "Patients Preference"[Title/Abstract:~4] OR "Patients Preferences"[Title/Abstract:~4] OR "Service User Preference"[Title/Abstract:~4] OR "Service User Preferences"[Title/Abstract:~4] OR "Service Users Preference"[Title/Abstract:~4] OR "Service Users Preferences"[Title/Abstract:~4] OR "thematic analys*"[Title/Abstract] OR "Patient Opinion"[Title/Abstract:~4] OR "Patient Opinions"[Title/Abstract:~4] OR "Patients Opinion"[Title/Abstract:~4] OR "Patients Opinions"[Title/Abstract:~4] OR "Service User Opinion"[Title/Abstract:~4] OR "Service User Opinions"[Title/Abstract:~4] OR "Service Users Opinion"[Title/Abstract:~4] OR "Service Users Opinions"[Title/Abstract:~4] OR "Patient Evaluation"[Title/Abstract:~4] OR "Patient Evaluations"[Title/Abstract:~4] OR "Patients Evaluation"[Title/Abstract:~4] OR "Patients Evaluations"[Title/Abstract:~4] OR "Service User Evaluation"[Title/Abstract:~4] OR "Service User Evaluations"[Title/Abstract:~4] OR "Service Users Evaluation"[Title/Abstract:~4] OR "Service Users Evaluations"[Title/Abstract:~4] OR "thematic synthes*"[Title/Abstract] OR "grounded theor*"[Title/Abstract] OR "Subjective experience"[Title/Abstract] OR "Subjective Experiences"[Title/Abstract]

**Appendix B**

**Table B.1** Reasons for excluded studies

| **Title** | **Year** | **Authors** | **Reason for exclusion** |
| --- | --- | --- | --- |
| Attempting to discontinue antipsychotic medication: Withdrawal methods, relapse and success. | 2018 | Larsen-Barr M and Seymour F and Read J and Gibson K | wrong/missing diagnosis |
| The lived experience of clozapine discontinuation in patients and carers following suspected clozapine-induced neutropenia. | 2023 | Oloyede E and Dunnett D and Taylor D and Clark I and MacCabe JH and Whiskey E and Onwumere J | other outcome |
| Perception towards relapse and its predictors in psychosis patients: A qualitative study. | 2018 | Hui CLM and Lo MCL and Chan EHC and Chen ESM and Ko RWT and Lee EHM and Chang WC and Chan SKW and Chen EYH | other outcome |
| Discontinuing Antipsychotic Medication After Remission from First-Episode Psychosis: A Survey of Psychiatrists' Attitudes in Taiwan. | 2022 | Yen K and Liu CC and Lin YT and Chien YL and Hsieh MH and Liu CM and Hwang TJ and Liao WH and Hwu HG | quantitative data |
| Reducing Antipsychotic Medication Use in Nursing Homes: A Qualitative Study of Nursing Staff Perceptions. | 2018 | Simmons SF and Bonnett KR and Hollingsworth E and Kim J and Powers J and Habermann R and Newhouse P and Schlundt DG | other outcome |
| Facilitators and Barriers Influencing Antipsychotic Medication Prescribing and Deprescribing Practices in Critically Ill Adult Patients: a Qualitative Study. | 2023 | Jaworska N and Krewulak KD and Schalm E and Niven DJ and Ismail Z and Burry LD and Leigh JP and Fiest KM | Wrong/missing diagnosis |
| Experiencing antipsychotic discontinuation: results from a survey of Australian consumers. | 2014 | Salomon C and Hamilton B and Elsom S | wrong/missing diagnosis |

**Appendix C**

**Table C.1** Complete CASP items

| **Study** | **1.Was there a clear statement of the aims of the research?** | **2. Is a qualitative methodology appropriate?** | **3.Was the research design appropriate to address the aims of the research?** | **4. Was the recruitment strategy appropriate to the aims of the research?** | **5. Was the data collected in a way that addressed the research issue?** | **6. Has the relationship between researcher and participants been adequately considered?** | **7. Have ethical issues been taken into consideration?** | **8. Was the data analysis sufficiently rigorous?** | **9. Is there a clear statement of findings?** | **10. How valuable is the research?** |
| --- | --- | --- | --- | --- | --- | --- | --- | --- | --- | --- |
| Cooper et al., 2019 | yes | yes | yes | yes | yes | yes | yes | yes | yes | yes |
| Crellin et al., 2022 | yes | yes | yes | yes | yes | not reported | yes | no | yes | yes |
| Gates et al., 2024 | yes | yes | yes | yes | yes | yes | yes | yes | yes | yes |
| Le Geyt et al., 2016 | yes | yes | yes | yes | yes | not reported | yes | yes | yes | yes |
| Lewins et al., 2024 | yes | yes | yes | yes | yes | yes | yes | yes | yes | yes |
| Morant et al., 2023 | yes | yes | yes | yes | yes | not reported | yes | yes | yes | yes |
| Nøstdal et al., 2024 | yes | yes | yes | yes | yes | not reported | yes | no | yes | yes |
| Roed et al., 2023 | yes | yes | yes | yes | yes | not reported | yes | yes | yes | yes |
| Southern et al., 2023 | yes | yes | yes | yes | yes | yes | yes | yes | yes | yes |
| Orlando et al., 2025 | yes | yes | yes | yes | yes | yes | yes | yes | yes | yes |

**Table C.2** Complete CASP comments

| **Authors** | **Item 1** | **Item 2** | **Item 3** | **Item 4** | **Item 5** | **Item 6** | **Item 7** | **Item 8** | **Item 9** | **Item 10** |
| --- | --- | --- | --- | --- | --- | --- | --- | --- | --- | --- |
| Cooper et al., 2019 | As part of preparation for a randomised controlled trial of antipsychotic reduction, we asked mental health professionals in secondary care about their attitudes towards, experiences of and the processes around antipsychotic reduction and discontinuation | A qualitative methodology is appropriate to conduct an exploratory study in preparation of a RCT | Focus groups are appropriate to explore clinicians' point of views | Participants were recruited from community-based mental health services and consisted of prescribing and non-prescribing mental health practitioners who were psychiatrists, mental health nurses, social workers, or clinical team managers. | Focus groups consisted of participants and facilitators only. Where possible, separate groups were conducted for psychiatrists and other clinical team members, in order to facilitate open discussion from the perspectives of prescribing and non-prescribing practitioners. Participants were also asked to complete a short demographics questionnaire. | Some participants were also known to the researchers due to participation in other research projects or clinical work. | The study was approved by the East of Scotland Research Ethics Service (Ref. 5/ES/0163). | Data were analysed using thematic analysis with NVivo software (Version 11) | Figure 1 | The findings are clearly discussed, and the sample is appropriately heterogeneous. |
| Crellin et al., 2022 | To explore the views of people with schizophrenia and other psychotic disorders about continuing their antipsychotic medication or attempting to reduce or discontinue this medication with clinical support | Quantitative and qualitative data were collected to explore patients' views | We conducted face-to-face interviews with people with a diagnosis of schizophrenia spectrum condition using a combination of structured and open-ended questions. | Participants were recruited from community mental health services and primary care practices across areas of London between April 2016 and August 2017. | Trained researchers administered a pre-designed interview schedule, including open and closed questions. The interviews were typically between 30 and 60 minutes in duration. Participants were given the opportunity to respond to an open question first and then presented with a series of fixed-format, mutually exclusive options and asked to decide. | NR | Ethical approval was provided by the East of Scotland Research Ethics Service (Research Ethics Committee reference: 15/ES/0163). | Only quantitative analysis was conducted | Table 3 | Although a thematic analysis was not conducted, illustrative quotes for each category are provided. |
| Gates et al., 2024 | The aim of this study was to explore the experience of consenting to and participating in a randomized controlled trial (RCT) of antipsychotic medication cessation. | Qualitative methodology is appropriate to explore personal experience of RCT participants | Participants completed a semi-structured qualitative interview | The participants who consented to the Reduce trial and finished trial participation were invited to participate in a follow-up qualitative interview conducted from April to June 2021. | Interviews were 30–90 minutes and conducted via online video call, which were audio recorded and transcribed. | JG was a Reduce trial clinician but also responsible for qualitative interviews, so only participants allocated to other Reduce trial clinicians were offered the opportunity to participate in the follow up to avoid bias and ethical considerations. | The participants who consented to the Reduce trial and finished trial participation were invited to participate in a follow-up qualitative interview conducted from April to June 2021. | Interpretive Phenomenological Analysis (IPA) was chosen for qualitative data analysis as it focuses on obtaining an in-depth understanding of how people make sense of experiences. | Table 3 | This research is valuable because it illuminates how young people with FEP experience antipsychotic dose reduction/cessation and RCT participation. |
| Le Geyt et al., 2016 | We conducted this study to explore personal accounts of making choices about taking medication prescribed for the treatment of psychosis (neuroleptics). | Qualitative methodology is appropriate to explore personal accounts | We then arranged a face-to-face interview at a location convenient to the participant and obtained written consent from them to participate. | We recruited between May 13 and August 23, 2014 in the North West of England. | We then arranged a face-to-face interview at a location convenient to the participant and obtained written consent from them to participate. | NR | We obtained ethical approval from the United Kingdom NHS ethics committee and the Research and Development departments of four NHS Trusts in the North West of England. | We used a grounded theory approach to analyse transcripts. | Findings are clearly divided into paragraphs and discussed accordingly | This research is valuable because it shows how people with psychosis actively weigh the costs and benefits of neuroleptics and gradually become experts in their own treatment. |
| Lewins et al., 2024 | To explore family members’ perspectives on antipsychotics, particularly their views on long-term use, reduction, and discontinuation of antipsychotics. | Qualitative methodology is appropriate to explore caregivers' perspectives on antipsychotics | A semi-structured interview topic guide was adapted from a focus group guide designed for mental health professionals that was developed in collaboration with service users and family members. | Participants were identified and recruited through a local carer support group and from the clinical caseloads of community mental health services. | Audio recordings were transcribed verbatim and anonymized | The first author was independent of the trial, and participants and their loved ones were not involved in the trial. | Ethical approval was obtained from the London-Brent Research Ethics Committee (ref:16/LO/1507). | Data were analysed using thematic analysis | Findings are clearly divided into paragraphs and discussed accordingly | This research is valuable because it reveals how family members balance fear of relapse with concerns about adverse effects, often prioritizing “fragile stability” through ongoing medication. |
| Morant et al., 2023 | The current study explored participants’ experiences of antipsychotic reduction or discontinuation within this trial. | Qualitative methodology is appropriate to explore the experiences of participants in a dose-reduction RCT | Semi-structured interviews, lasting 30–90 min, were conducted after the trial final 24-month follow-up with 26 people who reduced and/or discontinued antipsychotics within the trial. | Participants were sampled purposively from the RADAR trial intervention group to obtain a diverse sample. | Reflexive summary notes were made after each interview and shared among the team to inform data collection and analytic processes. Interviews were audio-recorded, transcribed and anonymised. | NR | Ethics approval was obtained (London-Brent Ethics Committee, 16/LO/1507). All participants provided informed consent. | Data were analysed using codebook thematic analysis within NVivo software. | Findings are clearly divided into paragraphs and discussed accordingly | This research is valuable because it shows, in real-world trial conditions, how people experience clinician-guided antipsychotic dose reduction over two years. |
| Nøstdal et al., 2024 | This study aimed to provide data on motivations for and previous experiences with antipsychotic tapering among patients attending the clinic. | An open-ended survey is appropriate to explore the experience of tapering among patients | Patients completed an open-ended survey on their motivations for discontinuing or tapering antipsychotic medication and recorded their expectations about these outcomes. | Patients were referred to the specialist outpatient clinic primarily from general practitioners and public health care outpatient clinics in the Capital Region of Denmark. | On enrolment, participants were asked to complete a questionnaire regarding their expectations about the outcomes of dose reduction or discontinuation of antipsychotics and a survey exploring their motivations for and previous experiences with antipsychotic dose reduction or discontinuation. | NR | The project protocol was submitted to the Danish National Committee on Health Research Ethics and the Danish Medicines Agency, both of which exempted the project from submitting a formal application because its scope and aims did not include examining the treatment effects, adverse effects, pharmacokinetics, or pharmacodynamics of antipsychotic medication and because the decision to reduce or discontinue antipsychotic medication was independent of any research results. | Only quantitative analysis was conducted | Box 1 | This research is valuable because it clarifies why many people with schizophrenia seek to taper or stop antipsychotics—despite past relapses—and how strongly they believe in the possibility of doing so successfully. |
| Roed et al., 2023 | This research is valuable because it reveals how mental health staff understand and position themselves in relation to antipsychotic tapering, highlighting the attitudes, uncertainties, and professional roles that shape real-world implementation. These insights can inform more coherent, collaborative, and patient-centred tapering practices. | Focus groups are appropriate to explore clinicians' experiences about tapering | Six audio-recorded focus groups were conducted. | Participants were recruited in mental healthcare services of two Danish regions. | Focus groups lasted between 45 and 77 minutes with a total of 6 hours and 44 minutes. Recordings were transcribed verbatim by a secretary. | NR | The study and data management plan were approved by the Capital Region’s data protection agency, Pactius (P-2022-169). | The data analysis combined an analysis of interactions between participants during focus groups with an analysis of the thematic content. | The findings are clearly presented as three distinctive positions (e.g., "Yes, we need to welcome risks...") | This research is valuable because it uncovers how mental health staff position themselves toward antipsychotic tapering—from risk-averse to recovery-oriented—showing how these stances can limit or enable patients’ requests to reduce medication. |
| Southern et al., 2023 | This study is the first of its type, seeking to explore people’s perspectives on clozapine discontinuation. | Semi-structured interviews are appropriate to explore patients' perspectives on clozapine discontinuation | Semi-structured interviews with sixteen patients who had received clozapine and discontinued treatment—thirteen males and three females, age range: thirty-two to seventy-eight years old—were audio-recorded and transcribed. | Potential participants were identified from a large secondary care mental healthcare organisation in North West England. | The interviews were audio recorded, transcribed verbatim, and anonymized by the researcher after each interview. | Where appropriate, for safety or participant assurance, care coordinators or relatives were in attendance for the interview. On two occasions, care coordinators were present, and one participant chose to have a relative present for support. | Approval for the study, which interviewed potentially vulnerable people about their treatment, was obtained from the Health Research Authority/NHS Ethics and Aston University Governance Committee. | This study’s methods had a basis in grounded theory, as defined by Charmaz 2014 | Findings are clearly divided into paragraphs and discussed accordingly | This research is valuable because it sheds light on how people experience stopping clozapine—often seen as the “best” option—and how this shapes their emotions, sense of control, and future treatment choices. |
| Orlando et al., 2025 | The aim of this study is to explore family and informal carers’ views and experiences of antipsychotic reduction and discontinuation within a medication reduction research trial (Research into antipsychotic Discontinuation and Reduction [RaDaR]). | Semi-structured interviews are appropriate to explore caregivers' perspectives on dose reduction and discontinuation | Participants were family members and informal carers of people randomised to the reduction/discontinuation arm of RADAR who had completed the trial within the previous year. | They were recruited from research sites in London. | Interviews lasted 45–90 min (except for one, lasting 23 min) and were audio-recorded with participants’ permission. | First author SO was independent of the trial and RS was a consultant with lived experience of family caring. | Ethical approval, including for minor pandemic-related amendments, was grantedby the London – Brent Research Ethics Committee [ref:16/LO/1507]. | Interviews were transcribed by study team members and anonymised | Table 3 | This research is valuable because it highlights the often-overlooked perspectives of family and informal carers on antipsychotic reduction, helping clinicians make more informed, collaborative decisions about medication changes and support. |

**Table C.3** Original themes reported in each included study

| **Study** | **Reported themes** |
| --- | --- |
| (Cooper et al., 2019) | Attitudes to antipsychotics and reduction; Reduction and discontinuation of antipsychotics in practice; Barriers to reducing antipsychotics |
| (Crellin et al., 2022) | Reasons for valuing or accepting long-term antipsychotic medication; Concerns about long term use of antipsychotics and reasons for wanting to reduce or stop; Factors that might facilitate antipsychotic reduction or discontinuation |
| (Gates et al., 2024) | Rejection versus identification with psychosis; Medication as symbolic of illness versus wellness; Embodiment of wellness and illness with medication; Medication as symbolic of independence versus dependence; Discovery of independence when autonomously choosing medication; Reduce offered safety to navigate the liminal space of FEP; Self-exploration versus altruism |
| (Geyt et al., 2017) | Developing a personal theory of the need for, and acceptability of, neuroleptic medication; Negotiating the challenge of forming alliances with others; Weaving a safety net to safeguard well-being |
| (Lewins et al., 2024) | Fragile stability; Ambivalence; Constant vigilance vs. autonomy |
| (Morant et al., 2023) | Effects of antipsychotic reduction/discontinuation; Making sense of reduction experiences: the RADAR trial as a novel potential learning context |
| (Nøstdal et al., 2024) | NR |
| (Orlando et al., 2025) | Starting points: pre-trial views and experiences; Perceived impacts of antipsychotic reduction/discontinuation; family/informal carer challenges during the trial; recommendations about carer support for antipsychotic reduction/discontinuation |
| (Roed et al., 2023) | Patients Will Eventually Realize That They Need the Medication; Tapering Means Running a Big Risk of Relapse in Symptoms; We Need to Welcome Risks to Support Personal Recovery |
| (Southern et al., 2023) | Positive and negative experiences of treatment; Feelings of agency; Feelings about future treatment |

**Appendix D**

**ENTREQ Checklist**

| N. | Item | Guide and description | Page |
| --- | --- | --- | --- |
| 1 | Aim | State the research question the synthesis addresses. | 3, section 1 |
| 2 | Synthesis methodology | Identify the synthesis methodology or theoretical framework which underpins the synthesis, and describe the rationale for choice of methodology (e.g. meta-ethnography, thematic synthesis, critical interpretive synthesis, grounded theory synthesis, realist synthesis, meta-aggregation, meta-study, framework synthesis). | 4, section 2.4 |
| 3 | Approach to searching | Indicate whether the search was pre-planned (comprehensive search strategies to seek all available studies) or iterative (to seek all available concepts until they theoretical saturation is achieved). | 3, section 2.1 |
| 4 | Inclusion criteria | Specify the inclusion/exclusion criteria (e.g. in terms of population, language, year limits, type of publication, study type) | 3, section 2.2 |
| 5 | Data sources | Describe the information sources used (e.g. electronic databases (MEDLINE, EMBASE, CINAHL, psycINFO, Econlit), grey literature databases (digital thesis, policy reports), relevant organisational websites, experts, information specialists, generic web searches (Google Scholar) hand searching, reference lists) and when the searches conducted; provide the rationale for using the data sources. | 3, section 2.1 |
| 6 | Electronic Search strategy | Describe the literature search (e.g. provide electronic search strategies with population terms, clinical or health topic terms, experiential or social phenomena related terms, filters for qualitative research, and search limits). | 3, section 2.1 + Appendix A |
| 7 | Study screening methods | Describe the process of study screening and sifting (e.g. title, abstract and full text review, number of independent reviewers who screened studies). | 3-4, section 2.3 |
| 8 | Study characteristics | Present the characteristics of the included studies (e.g. year of publication, country, population, number of participants, data collection, methodology, analysis, research questions). | 6, Table 1 |
| 9 | Study selection results | Identify the number of studies screened and provide reasons for study exclusion (e,g, for comprehensive searching, provide numbers of studies screened and reasons for exclusion indicated in a figure/flowchart; for iterative searching describe reasons for study exclusion and inclusion based on modifications t the research question and/or contribution to theory development). | 5, figure 1 + appendix B |
| 10 | Rationale for appraisal | Describe the rationale and approach used to appraise the included studies or selected findings (e.g. assessment of conduct (validity and robustness), assessment of reporting (transparency), assessment of content and utility of the findings). | 3, section 2.3 |
| 11 | Appraisal items | State the tools, frameworks and criteria used to appraise the studies or selected findings (e.g. Existing tools: CASP, QARI, COREQ, Mays and Pope; reviewer developed tools; describe the domains assessed: research team, study design, data analysis and interpretations, reporting). | 3-4, section 2.3 + appendix C |
| 12 | Appraisal process | Indicate whether the appraisal was conducted independently by more than one reviewer and if consensus was required. | 3-4, section 2.3, section 2.4 |
| 13 | Appraisal results | Present results of the quality assessment and indicate which articles, if any, were weighted/excluded based on the assessment and give the rationale. | 11, section 3.2 |
| 14 | Data extraction | Indicate which sections of the primary studies were analysed and how were the data extracted from the primary studies? (e.g. all text under the headings “results /conclusions” were extracted electronically and entered into a computer software). | 4, section 2.4 |
| 15 | Software | State the computer software used, if any. | 4, section 2.4 |
| 16 | Number of reviewers | Identify who was involved in coding and analysis. | 4, section 2.4 |
| 17 | Coding | Describe the process for coding of data (e.g. line by line coding to search for concepts). | 4, section 2.4 |
| 18 | Study comparison | Describe how were comparisons made within and across studies (e.g. subsequent studies were coded into pre-existing concepts, and new concepts were created when deemed necessary). | 4, section 2.4 |
| 19 | Derivation of themes | Explain whether the process of deriving the themes or constructs was inductive or deductive. | 4, section 2.4 |
| 20 | Quotations | Provide quotations from the primary studies to illustrate themes/constructs, and identify whether the quotations were participant quotations of the author’s interpretation. | 8-9, section 3.1 |
| 21 | Synthesis output | Present rich, compelling and useful results that go beyond a summary of the primary studies (e.g. new interpretation, models of evidence, conceptual models, analytical framework, development of a new theory or construct) | 11-12, section 4 |
